# Fibrillated Films for Suspension Catalyst Immobilization—A Kinetic Study of the Nitrobenzene Hydrogenation

**DOI:** 10.3390/ma17225411

**Published:** 2024-11-06

**Authors:** Chiara Boscagli, Enrico Lepre, Oliver Hofmann, Lukas Wengeler, Marcel Schmitt, Ivana Jevtovikj, Carlos Lizandara-Pueyo, Stephan A. Schunk

**Affiliations:** 1 hte GmbH, 69123 Heidelberg, Baden Wuerttemberg, Germany; 2 Group Research, BASF SE, 67059 Ludwigshafen, Rhineland-Palatinate, Germany; 3 Institute of Chemical Technology, Universität Leipzig, Linnéstr. 3, 04103 Leipzig, Germany

**Keywords:** fibrillated films, catalysis, hydrogenation

## Abstract

The immobilization of suspension catalysts in flexible, fibrillated films offers a promising solution to the mass transfer limitations often encountered in three-phase hydrogenation reactions. This study investigates the catalytic performance and mass transfer properties of fibrillated films in the hydrogenation of nitrobenzene to aniline, comparing them to free-flowing powdered catalysts. Fibrillated films were prepared from Pd/C catalysts with varying thicknesses (100–400 µm), and their performance was evaluated through kinetic studies in both batch reactors and microreactors. The specific activity of the films was significantly influenced by film thickness with thinner films demonstrating lower mass transfer limitations. However, mass transfer limitations were observed in thicker films, prompting the development of alternative film designs, including enhanced macro-porous films and sandwich structures. These modifications successfully minimized diffusion limitations, achieving similar specific activity to the powder catalysts while maintaining the mechanical stability of the films. This work demonstrates the feasibility of using fibrillated films for continuous catalytic processes and highlights their potential for efficient catalyst reuse, avoiding filtration steps and enhancing process sustainability. Furthermore, while PTFE remains indispensable for producing such films due to its mechanical and thermal stability, ongoing research focuses on identifying more environmentally friendly alternatives without compromising performance.

## 1. Introduction

Catalyst shaping is a fundamental step in industrial catalysis, often involving the transformation of powders into shaped bodies such as tablets, extrudates, or granules. While shaping enhances mechanical stability and facilitates catalyst handling, it can introduce challenges such as internal mass transfer limitations and a reduction in the available catalytic surface area. These limitations are particularly critical in three-phase reactions, where efficient mass transfer between gas, liquid, and solid phases is essential. Traditional shaped catalyst bodies, being rigid and of fixed dimensions, may not always provide the flexibility required for optimal catalytic performance, especially when mass transfer is a key factor. To address these challenges, novel approaches that enable the shaping of catalysts without compromising their activity are of growing interest [1,2,3,4,5,6,7,8,9,10,11,12,13].

Fibrillated films represent one such approach, offering flexibility in catalyst shape, thickness, and porosity. Composed of inorganic particles embedded within a nanofibrous polymer network, these films maintain high surface-to-volume ratios and internal porosity, minimizing mass transfer limitations while preserving the catalytic properties of the original powder. Furthermore, their mechanical flexibility allows for their use in both batch and continuous flow processes, presenting a significant advantage over rigid catalyst supports [14,15]. Moreover, fibrillated films can serve as supports for various catalytically active materials such as metal nanoparticles or catalysts supported on polymer particles. The perspective is that such active components supported on particulate supports can be deposited into the nanofibrous structure [16], preserving a high number of catalytic sites and therefore allowing a high intrinsic catalytic activity for a large number of reactions [2].

Polytetrafluoroethylene (PTFE) is frequently used to produce fibrillated films due to its excellent chemical and thermal stability [17,18]. Despite concerns about the environmental impact of fluorinated compounds, PTFE remains critical in many catalytic and electrochemical applications, providing durability under harsh reaction conditions such as elevated temperatures or corrosive environments. While alternatives to PTFE are under investigation, its unique properties make it difficult to replace without sacrificing performance. PTFE it is widely used and indispensable as a binder in electrocatalyst preparation [19,20,21]. Moreover, examples were presented where PTFE was used as a fibrillating agent in air filters for catalysts enabling the conversion of volatile organic compounds into harmless species, such as CO_2_ and water [22,23]. A similar technology approach is also employed in the production of honeycomb-structured catalysts for the treatment of large amounts of gas at high linear flow rates, such as the decomposition of NOx [24,25].

Wristers et al. described a method involving the mixing of solid catalyst particles with a fibrillable PTFE polymer between 0.01 and 5 wt.% to create shaped catalysts [26]. Similarly, Bernstein et al. showed a method for fabricating a polymeric catalyst structure comprising a particulate catalyst material enclosed in a porous, fiber-containing polymeric material, incorporating 1 to 5 wt.% of a fibrillated PTFE polymer [27]. The same authors prepared a catalyst suitable for fixed or fluidized catalyst beds based on an active or activatable material, a fibrillated first polymer, and a second polymer that contributes to the support [28]. Moreover, the usage of fibrillated films as catalysts was shown to be of interest for academic endeavors. Chen Yijun et al. describe the preparation of Au nanocrystals using alpha-zein in fibrillated form [14]. Renliang Huang et al. presented a catalytic membrane reactor composed of a membrane matrix and a catalytic film consisting of alloy nanoparticle-loaded protein fibrils. This membrane reactor was used for the continuous reduction of 4-nitrophenol [16]. Specifically, the reduction of nitrobenzene and its derivates is a reaction attracting attention for producing important intermediates for dyes, pharmaceuticals, and pesticides [29,30]. Therefore, it was selected as a model reaction for the test of fibrillated film catalysts. This study explores the application of fibrillated films for immobilizing suspension catalysts in hydrogenation reactions, focusing on the hydrogenation of nitrobenzene to aniline as a model reaction. Nitrobenzene hydrogenation is an industrially relevant reaction, producing key intermediates for pharmaceuticals, dyes, and agrochemicals. The high kinetics of this reaction make it a suitable probe for investigating mass transfer phenomena, particularly in heterogeneous catalysis. The primary goal of this work is to compare the catalytic activity and mass transfer properties of fibrillated films with those of free-flowing powder catalysts and to explore methods for enhancing film performance by modifying film structure. Specifically, we introduce two film designs: (i) sandwich films, where active catalytic layers are confined to the surface regions of the film, and (ii) enhanced macro-porous films, which increase reagent access to active sites. These modifications aim to mitigate the mass transfer limitations observed in thicker films, improving catalytic efficiency. The findings of this study showcase the incredible potential of fibrillated films to deliver outstanding catalytic performance while effectively overcoming mass transfer limitations. These innovative films bring even more advantages, such as enhanced catalyst reusability, effortless handling, and ideal compatibility for continuous processing in microreactors. This research marks a significant step forward in creating advanced catalyst supports that blend high activity with the practical benefits of shaped bodies, unlocking exciting new possibilities for industrial applications.

## 2. Results and Discussion

### 2.1. Preparation and Characterization of the Fibrillated Films

Fibrillated films were formed by mixing commercial 5 wt.% Pd/C catalyst powder with 7.5 wt.% PTFE binder. The mixture was subjected to mechanical treatments including kneading and calendaring to form films of varying thicknesses (100 µm, 250 µm, and 400 µm) (refer to Supplementary Materials Section S1). Three different film thicknesses were prepared for each film: 100 µm, 250 µm, and 400 µm.

**Figure 1:**
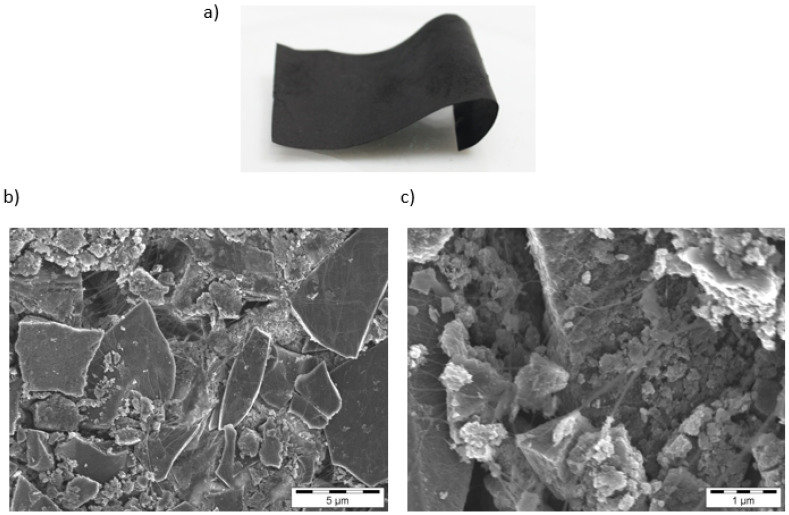
Fibrillated film image (**a**); SEM image of the 100 µm fibrillated film produced from the high activity Pd/C at different magnifications (**b**,**c**).

Table 1 summarizes the key physical properties of the Pd/C catalysts used, while Table 2 provides details of the fibrillated films. The structure of the fibrillated films was analyzed using SEM (Figure 1), which revealed that PTFE nanofibrils effectively bind the carbon grains, preserving the internal structure of the catalyst. The pore size distributions and surface areas of the films were found to decrease with decreasing film thickness, which is attributed to the calendaring process. The BET surface areas of the fibrillated films decreased with increasing film thickness with the 100 µm film showing a surface area of 604 m^2^/g compared to 764 m^2^/g for the powder. This reduction in surface area is critical as it directly impacts the mass transfer properties of the films, especially in reactions where internal diffusion is a limiting factor. Despite this, the fibrillated films maintain sufficient porosity to facilitate catalyst accessibility, as confirmed by nitrogen sorption measurements.

### 2.2. Catalytic Performance and Mass Transfer Limitations in Batch Reactors

**Figure 2:**
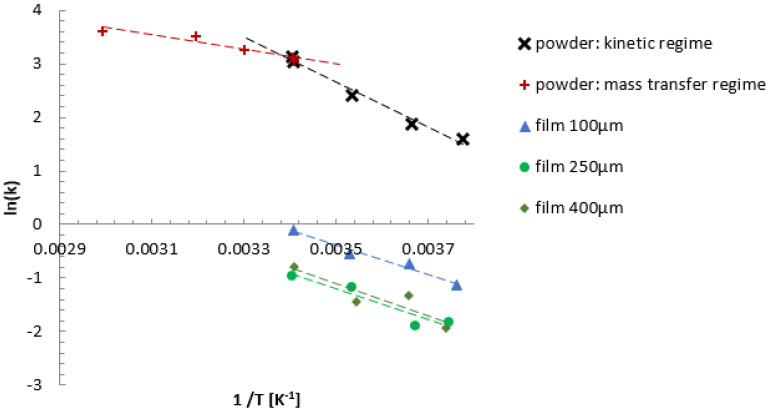
Arrhenius plot for high activity Pd/C catalyst as powder and fibrillated film with thickness 100, 250, 400 µm (temperature range −8–20 °C for the fibrillated films, H_2_ pressure 5 barg, nitrobenzene concentration 0.029 mol·L^−1^). k is calculated and reported for zero order reaction.

The catalytic performance of the fibrillated films was evaluated in the hydrogenation of nitrobenzene to aniline, which is a reaction commonly used to assess mass transfer limitations due to its fast kinetics [31]. Hydrogen consumption data were collected in a batch autoclave, and the reaction rates were normalized per gram of palladium. The powder catalyst exhibited high reaction rates, with minimal mass transfer limitations, as evidenced by the near-linear Arrhenius plot between −8 and 20 °C (Figure 2).

**Figure 3:**
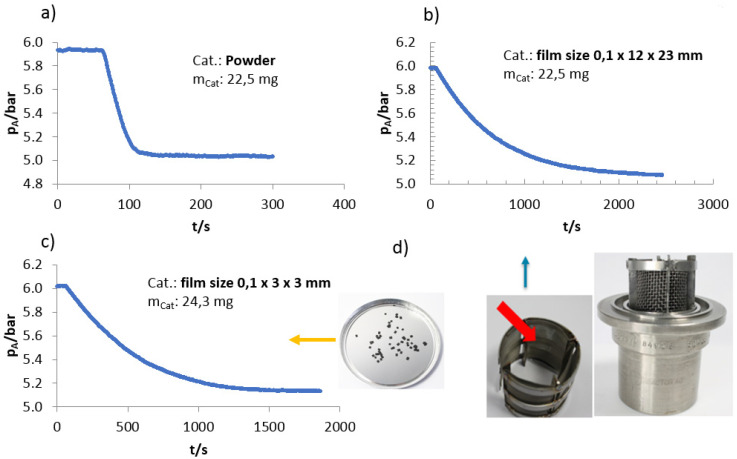
Hydrogen pressure consumption in the autoclave over time. Different forms of the high activity Pd/C were used: (**a**) powder, (**b**) fibrillated film size 0.1 × 12 × 33 mm, (**c**) fibrillated film size 0.1 × 3 × 3 mm. Reaction condition: 20 °C, H_2_ pressure 5 barg, 0.03 mol/L nitrobenzene, (**d**) Pictures of the fibrillated films used in (**b**) (indicated by the orange arrow) and (**c**) (indicated by the blue arrow). The red arrow indicates the position of the fibrillated film in the reactor.

However, the fibrillated films, particularly those with higher thicknesses (250 µm and 400 µm), showed significant mass transfer limitations. The effectiveness factor for the 400 µm film was less than 8%, highlighting severe internal diffusion limitations (see Figure S3, Supplementary Materials). In contrast, the 100 µm film exhibited an effectiveness factor of up to 47%, suggesting better accessibility to active sites, although it was still lower than the powder catalyst. The lower reaction rates for the fibrillated films can be attributed to restricted mass transfer, as thicker films hinder the efficient diffusion of reactants to the catalyst’s active sites. This limitation was further confirmed by the shift in reaction order for the fibrillated films, which ranged between 0.5 and 1, compared to the zero-order kinetics observed for the powder catalyst under the same conditions (Figure 3). The reaction order shift indicates that mass transfer constraints are playing a significant role in controlling the overall rate of the reaction.

### 2.3. Strategies to Improve Mass Transfer: Sandwich and Macro-Porous Films

In response to the observed mass transfer limitations, two strategies were implemented to enhance the performance of the fibrillated films: (i) sandwich films, in which the active catalyst is concentrated in the outer layers, and (ii) macro-porous films, which increase the accessibility of reactants through larger pore structures.

**Figure 4:**
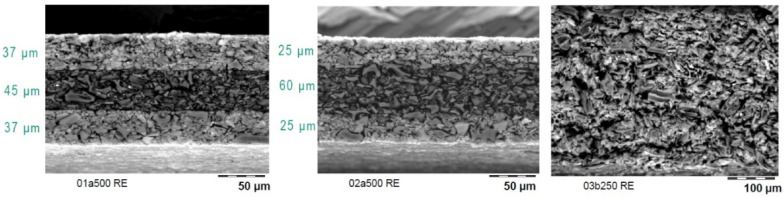
SEM images showcasing the vertical profile of sandwich films created by compressing either thin (left image) or thick films (middle image). The image on the right displays the film prior to compression. For these observations, the film was sliced using Focused Ion Beam (FIB) technology and analyzed in backscattering mode. The bright regions indicate the presence of Pd (palladium).

**Figure 5:**
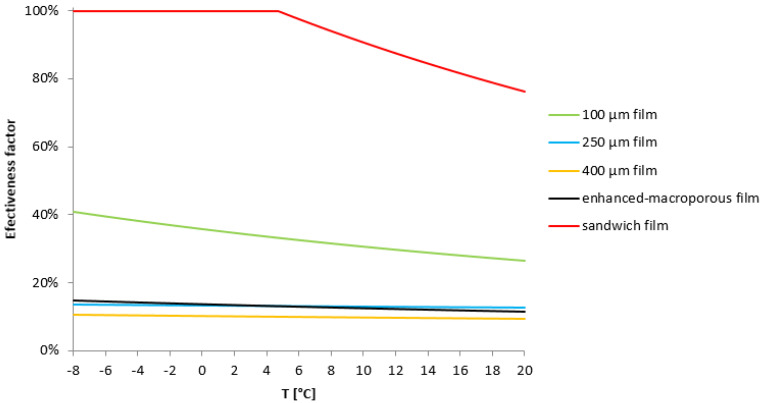
Effectiveness factor of the fibrillated film produced from low-activity Pd/C catalyst in different thickness, as sandwich film (thickness 100 µm) and as porous film (enhanced macro-porous film, thickness 250 µm).

Sandwich films were prepared by positioning the Pd/C catalyst in the outer layers of the film, creating a structure like eggshell catalysts. This design ensures that the catalyst is more readily accessible to reactants, reducing internal diffusion limitations. SEM imaging (Figure 4) confirmed the successful fabrication of sandwich films with an active catalytic layer thickness of approximately 31 µm. These films demonstrated a marked improvement in catalytic performance with an effectiveness factor approaching that of the free-flowing powder (Figure 5).

Macro-Porous Films: To further enhance mass transfer, macro-porosity was introduced into the fibrillated films by incorporating soluble agents (such as polyethylene oxide, PEO) that were subsequently leached out after film formation. The resulting macro-porous films exhibited larger pores, facilitating a more efficient diffusion of reactants. The 250 µm macro-porous films demonstrated improved catalytic activity compared to their non-porous counterparts, as evidenced by their higher effectiveness factors (Figure 5). These results confirm that increasing porosity mitigates diffusion limitations without compromising mechanical stability.

### 2.4. Investigation of Reaction Orders and Rate Constants

The change in reaction orders observed for the fibrillated films, shifting from zero-order kinetics in the powder catalyst to fractional orders (0.5 to 1), further highlights the role of mass transfer in limiting the reaction rate. This change suggests that internal diffusion restrictions in the fibrillated films are impeding access to the active sites, particularly for the thicker films. Rate constants were calculated from the hydrogen consumption data, and the corresponding Arrhenius plot (Figure 2) shows a lower apparent activation energy for the fibrillated films compared to the powder catalyst. For the powder catalyst, the activation energy was found to be 33 kJ/mol, while the fibrillated films showed a lower average of 23 kJ/mol, indicating the presence of diffusion limitations, which is consistent with reactions operating in the kinetic regime but transitioning to mass transfer control at higher temperatures [32]. This supports our conclusion that the introduction of porosity and optimized film designs can shift the reaction back into the kinetic regime by alleviating diffusion constraints.

### 2.5. Performance in Microreactor Flow Systems

**Figure 6:**
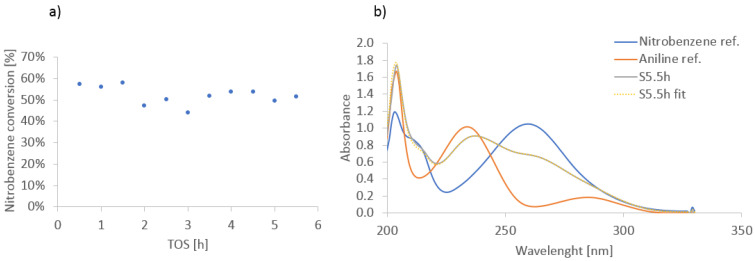
Nitrobenzene conversion recorded in microreactor at 20 °C and approximate space time 5 s (**a**). UV-Vis spectrum of a sample collected at TOS = 5.5 h (S 5.5 h) and its fit (S 5.5 h) using a linear combination of the nitro-benzene and aniline (**b**).

**Figure 7:**
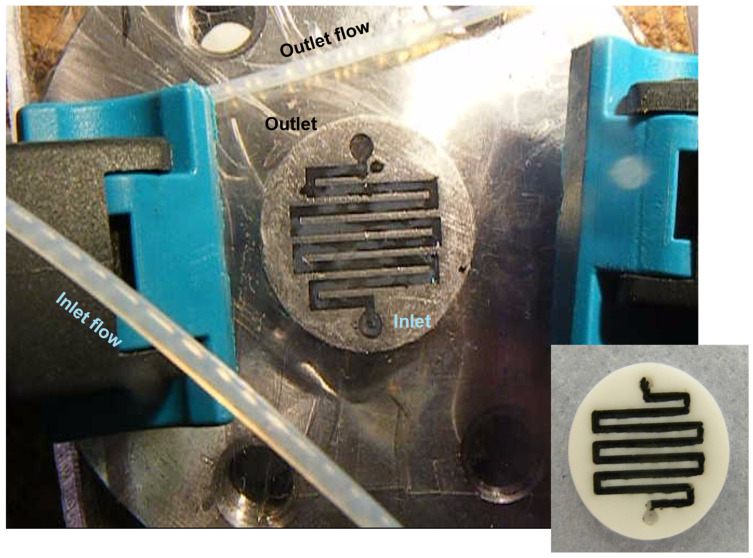
Microreactor channels with immobilized a fibrillated film. Taylor flow is observed at the inlet and outlet of the reactor and on the fibrillated film.

The performance of fibrillated films in continuous flow systems was evaluated in a microreactor setup with Taylor flow. A 100 µm thick film was immobilized in the microchannels, and the hydrogenation of nitrobenzene was performed at 20 °C. The conversion of nitrobenzene remained stable over a 5 h run (Figure 6 and Figure 7) with high selectivity (>99%) toward aniline.

This stable performance confirms the suitability of fibrillated films for continuous operation (Figure 7), where catalyst reusability and mechanical stability are critical. The mechanical integrity of the films remained intact, even under the high flow rates and agitation conditions, further demonstrating their potential for industrial-scale continuous flow applications. The stability and reusability of the fibrillated films were assessed through repeated reaction cycles. After four cycles, the catalytic activity decreased to 59% of the original value, which is consistent with the deactivation behavior observed for the powdered catalyst. SEM analysis after the reaction revealed no significant mechanical degradation of the films, suggesting that deactivation is primarily due to the typical deactivation mechanisms of a hydrogenation Pd catalyst, such as fouling or leaching, rather than the mechanical failure of the film structure [33].

## 3. Conclusions

This study powerfully demonstrates the transformative potential of fibrillated films as flexible and efficient supports for suspension catalysts, particularly in the hydrogenation of nitrobenzene to aniline. Key findings reveal that thicker fibrillated films (250 µm and 400 µm) face substantial mass transfer limitations, which are evidenced by the markedly reduced reaction rates and effectiveness factors. In contrast, the thinner 100 µm films, while outperforming their thicker counterparts, still encounter diffusion constraints when compared to the free-flowing powdered catalyst. These insights underscore the critical role of film thickness in optimizing catalytic performance, paving the way for future innovations in catalyst design and application. Two structural designs were introduced to address mass transfer limitations: sandwich films and macro-porous films. The sandwich films concentrated the active catalytic material near the surface, improving accessibility and enhancing catalytic performance. The macro-porous films introduced larger pore structures, which significantly improved mass transfer without compromising the mechanical integrity of the films.

Fibrillated films were successfully employed in continuous flow systems, demonstrating stable performance over prolonged operation. The films maintained high catalytic activity and selectivity (>99%) toward aniline, highlighting their suitability for continuous catalytic processes, such as those in microreactor applications.

While the fibrillated films exhibited excellent mechanical stability, catalyst deactivation was observed over repeated reaction cycles. The deactivation mechanisms are believed to be related to fouling or palladium leaching rather than mechanical degradation. Future work will focus on exploring regeneration strategies to prolong the operational lifetime of the films. Fibrillated films represent a versatile and scalable technology for catalytic applications with significant potential for continuous processing in both laboratory and industrial settings. The ability to tailor the film structure to mitigate mass transfer limitations opens new avenues for optimizing catalyst performance. Future research will explore alternative materials to replace PTFE and further improve the long-term stability and reusability of the films through advanced regeneration techniques. Additionally, expanding the application of fibrillated films to other catalytic reactions will provide further insights into their broader industrial relevance.

## Figures and Tables

**Table 1:** Physical and chemical characteristic of the three Pd/C catalysts.

	High Activity Pd/C	Middle Activity Pd/C	Low Activity Pd/C
Activity	high	Middle	Low
Pd loading	5%	5%	5%
BET surface area	764 m^2^/g	758 m^2^/g	789 m^2^/g
Pore volume	0.60 cm^3^/g	0.61 cm^3^/g	0.63 cm^3^/g
Grain size D_n10_	0.754 μm	0.728 μm	0.943 μm
D_n50_	1.05 μm	1.09 μm	1.44 μm
D_n90_	2.66 μm	3.07 μm	4.18 μm
Pd dispersion	23.6%	23.1%	29.6%
Pd surface area	5.3 m^2^/g	5.1 m^2^/g	6.6 m^2^/g
Pd particle size (hemisphere, chemisorption)	4.7 nm	4.8 nm	3.8 nm

**Table 2:** Physical and chemical characteristic of the fibrillated film produced from the powder Pd.

High Activity Pd/C	Powder	100 μm Thick Film	250 μm Thick Film	400 Thick Film
PTFE content	-	7.5%	7.5%	7.5%
Pd loading	5%	4.63%	4.63%	4.63%
BET surface area	764 m^2^/g	604 m^2^/g	654 m^2^/g	717 m^2^/g
Pore volume	0.60 cm^3^/g	0.47 cm^3^/g	0.50 cm^3^/g	0.55 cm^3^/g
Pd dispersion	23.6%	20.5%	23.8%	24.1%
Pd surface area	5.3 m^2^/g	4.2 m^2^/g	4.9 m^2^/g	5.0 m^2^/g
Pd particle size (hemisphere, chemisorption)	4.7 nm	5.5 nm	4.7 nm	4.6 nm

## Data Availability

The original contributions presented in the study are included in the article and Supplementary Materials, further inquiries can be directed to the corresponding authors.

## Supplementary Materials

The following supporting information can be downloaded at https://www.mdpi.com/article/10.3390/ma17225411/s1, Supporting information, such as additional information about the Experimental methods. Section S1.1: Materials and Devices; Section S1.2: Materials Synthesis, Section S1.3: Characterization; and Section S1.4: Catalytic Evaluation. Figure S1: Appearance of the film from the pre-fibrillated material to the film results. The middle image represents the status of the film after the light roller and before the heavy roller; Figure S2: Basic Autoclave Equation plot. In the graph, r _global_ indicates the apparent reaction rate and mPd is the absolute mass of Pd employed for each experiment; Figure S3: Effectiveness factor of the fibrillated films produced from the three Pd/C catalysts (see Table 1 and Table 2).
