# Supplementary material for: Fibrillated Films for Suspension Catalyst Immobilization—A Kinetic Study of the Nitrobenzene Hydrogenation
Source: Materials (Basel). 2024 Nov 6;17(22):5411. doi: 10.3390/ma17225411 (PMC11596000; doi:10.3390/ma17225411)
Supplement: Supplementary file 1 [file materials-17-05411-s001.zip › materials-3193898-supplementary.pdf]

Supplementary Materials

Fibrillated Films for Suspension Catalyst Immobilization - A Kinetic Study of the Nitrobenzene Hydrogenation

Chiara Boscagli[1+], Enrico Lepre[1+], Oliver Hofmann[1], Lukas Wengeler[2], Marcel Schmitt[2], Ivana Jevtovikj[1], Carlos Lizandara-Pueyo[2*], and Stephan A. Schunk[1,2,3*]

[1]*hte company GmbH*
Heidelberg, Baden Wuerttemberg, 69123, Germany

[2]*Group Research*
BASF SE
Ludwigshafen, Rhineland-Palatinate 67059, Germany

[3]*Institute of Chemical Technology, Universität Leipzig*
Linnéstr. 3, 04103 Leipzig, Germany

*Corresponding authors: *carlos.lizandara@basf.com*, *stephan.schunk@hte-company.de*

[+] Authors contributed equally to the work

1. Experimental methods

1.1 Materials and Devices

Methanol (purity > 99%) is used as a solvent, the palladium catalyst to be investigated on activated carbon and a nitrobenzene solution (purity > 99.9%).

To produce the films the following devices were used: Durston Rolling Mills Hand Rolling Mill (ABB1), Mitutoyo Film Thickness Gauge Model No.: ID-H0530 (ABB2), Laborwaage Sartorius AGCPA6202S, Rollbock RLabinco(ABB3), Hand rollers (2.3kg, wide/ 4.75kg, narrow) (ABB4/5), Steel balls, diameter: 0.8 cm, Carpet Knife Blade

1.2 Materials Synthesis

1.2.1 Synthesis of fibrillated films

The synthesis of the films was conducted through 3 stages: 1. Mixing, 2. Pre-fibrating, and 3. Film production.

The first mixing stage was performed to mix the activated carbon and binder well, both components are transferred to a cylindrical plastic container (2pprox.. 1.5 L). A piece of plastic tubing is glued into this container to achieve a circulation of the material. Each batch was mixed for at least one hour at Rollbock Level 2-3.

After mixing, the material must be pre-fibrillated. To do this, 750g of steel balls are added to 10g of powder, also in a cylindrical plastic container (1.5L). The process is completed when most of the material has settled to the bottom or lid of the jar (4-7 minutes). Then remove the steel ball by means of a sieve.

The pre-fibrillated material is laid out on the worktable and crushed with the carpet knife blade. The light roller (2.3 kg) is then rolled over the material without pressure until the material is no longer noticeably altered by the light roller. The edges are straightened with the box cutter blade. If there are many cracks in the film, it is cut in half in half, folded and worked again with the light roller.

The heavy roller is used when the film looks homogenous. It is rolled until the film no longer changes noticeably. Again, the edges are straightened, if there are cracks in the film, it is folded again.

Before using the hand rolling mill, the film should look something like Fig. 9. The layer thickness of the film is measured, and the rolling mill is set to a gap that is about 20% smaller than the film thickness. Subsequently, the gap is always reduced by 3 marks on the rolling mill scale, up to a thickness of 375 µm, then two marking lines up to 250 µm, then one marking line up to 125 µm. Underneath, no more than half a mark should be narrowed. From 125µm onwards, special care must be taken to ensure that it is only tightened when no resistance is felt when rolling.

During calendaring, cracks at the edges of the film are removed or cut off.

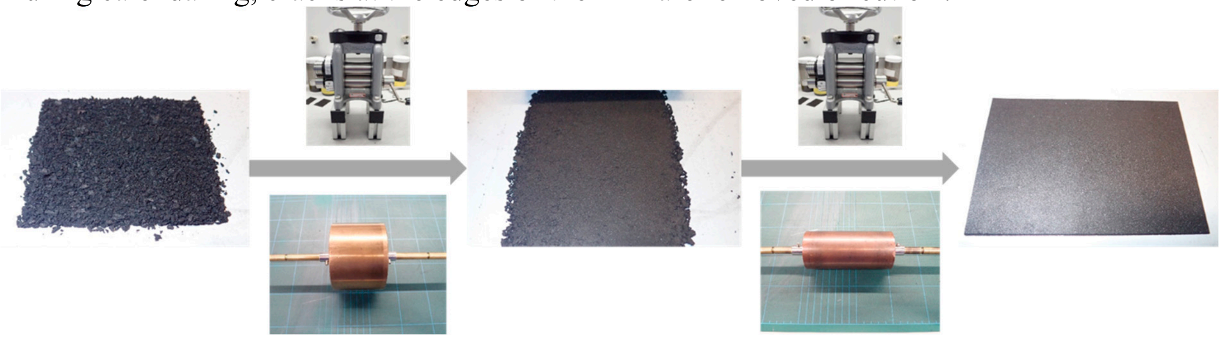

Figure S1: Appearance of the film from the pre-fibrillated material to the film results. The middle image represents the status of the film after the light roller and before the heavy roller.

1.2.2 Synthesis of porous fibrillated films

The development of porosity in the films was developed through the calendaring process. Specifically, by combining multiple layers of the same materials the porosity of the films was increased.

1.2.3 Synthesis of sandwich fibrillated films

The development of sandwich fibrillated films was obtained by the calendaring of films of different materials.

1.3 Characterization

The particle size distribution of the powder catalysts is measured using laser diffraction. The particles are dispersed in water and pumped through the measuring cell. The particle refractive index is 2.420 for carbon and the number distribution is determined.

The mesopore size of the powders and felts is examined by physisorption (BET isotherm, 100 measuring points, nitrogen).

Furthermore, metal dispersion is investigated by chemisorption of carbon monoxide (CO). For this purpose, the samples were first reduced with hydrogen (flux 10 ml) at 100 °C and then measured with the pulse chemisorption method at a flux of 50 ml-CO.

The powder catalysts were examined using TEM (transmission electron microscopy) and the felts were examined using SEM (scanning electron microscopy). For the TEM measurement, the sample was dispersed into ethanol and transferred to the measuring device (Tecnai G2-F20ST of the FEI Company, Hillsboro, USA). It was worked at a voltage of 200 keV. For the SEM images, a measuring device with a field emission emitter was used and images were taken at 3 kV.

1.4 Catalytic Evaluation

1.4.1 Batch reactor experiments

The autoclave reactions were carried out in a 60 ml autoclave (batch reactor) from Premex solutions GmbH. The autoclave is equipped with an overhead stirrer (max. rotational frequency 2000 min1, agitator: disc stirrer). The reaction was followed via consumption of the hydrogen using two methods. The first method considered the autoclave s a closed system and measured the reactor pressure decrease. The second method measured the amount of hydrogen fed to the autoclave via a burette to keep the reactor pressure constant.

> Standard test conditions: Test temperature: -8 to 60 °C, total pressure: 5 barg; Rotation frequency of stirrer: 2000 min^{-1}; Nitrobenzene concentration in the autoclave: 0.03 mol/L; Solvent in autoclave: 25 mL methanol; 5 mL nitrobenzene stock solution 0.18 mol/L in charger.

In a typical test methanol is pipetted into the 60 ml autoclave. The powder catalyst is dispersed into the methanol presented, while the catalyst felts are fixed to holders of the breaker before the methanol is added. The autoclave is then sealed pressure-tight, and 5 ml of nitrobenzene solution is pipetted into the charger. The entire apparatus must be sealed pressure tight.

After the apparatus has been prepared, the specified hydrogen atmosphere must be set. To do this, the system is flushed with a stream of nitrogen for 2 minutes, the first minute without stirring and the second minute with a stirrer rotation frequency of approx. 600 min^{-1}. The apparatus is loaded with approx. 35 barg nitrogen a total of five times to flush dead volumes of the apparatus as well. Finally, a total pressure of 5 barg of hydrogen is set at 20 °C. Under these conditions and a rotational frequency of the stirrer of 2000 min^{-1}, the passivated catalytic converter is reduced/activated for 10 minutes. By opening the charger, the reaction is initiated, and the course of the experiment is documented via the paperless graphic recorder (CHESSLLER recorder). The experiment ends as soon as the system is in mechanical and thermal equilibrium for at least 1 minute.

Once the test is complete, the autoclave is tempered to 20 °C. Only then should the system be relaxed and rinsed with nitrogen for at least 1 minute at a stirring frequency of approx. 600 min1. The relaxed and rinsed apparatus is opened, the lid of the autoclave is rinsed with water

and a sample is taken. All components contaminated with catalytic converters and methanol can spontaneously ignite and are therefore first rinsed with plenty of water. Subsequently, the entire apparatus is cleaned with ethanol. The entire plant must be brought to a clean and dry state before another experiment can be carried out.

1.4.2. Kinetic Experiments in Batch

For the kinetic studies, the following procedure was developed. The catalyst (Pd/C), solvent (methanol), and hydrogen were loaded into the autoclave at a pressure of 5 barg. A solution of nitrobenzene was inserted into the charger. The autoclave was then stabilized at the desired temperature.

The reaction began at t_0 when the valve of the charger was opened, and nitrobenzene solution was inserted into the reactor (resulting in a final nitrobenzene concentration of 0.03 mol/L). The consumption of hydrogen during the reaction was calculated by recording the pressure variation in the autoclave over time. Based on this, the amount of hydrogen consumed and the variation in nitrobenzene concentration were calculated.

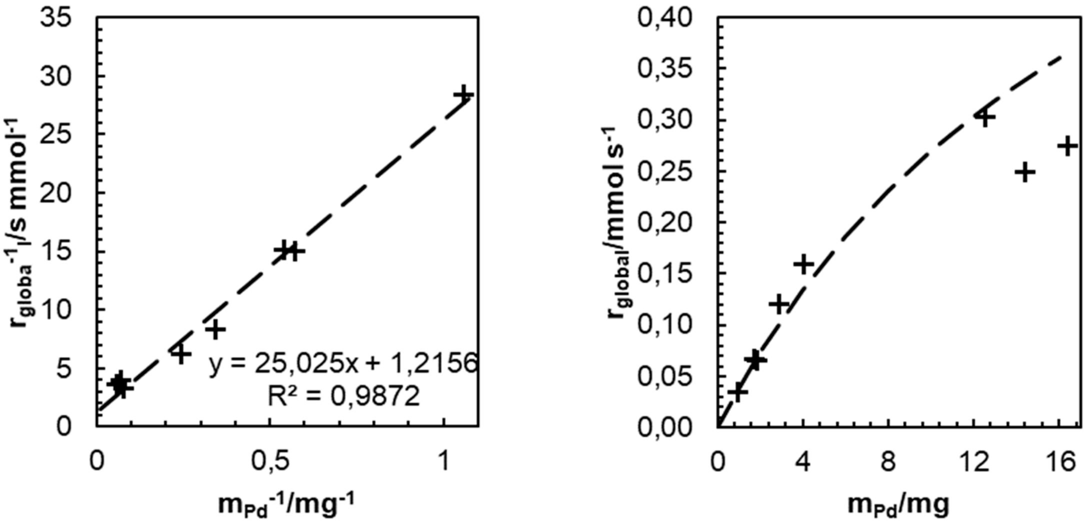

Figure S2. Basic Autoclave Equation plot. In the graph, r_{global} indicates the apparent reaction rate and mPd is the absolute mass of Pd employed for each experiment.

The reaction rate was reported in the form of mols^{-1} of converted nitrobenzene normalized for the mass unit of Palladium metal. The powder and fibrillated films were compared using Arrhenius plots and the respective effectiveness factors. The temperature range studied was

from -8 °C to 20 °C, where data for the free-flowing powder catalyst were obtained without apparent external or internal mass transport limitations; in this way it can be ensured that measurements were made in the kinetic regime and data that allow interpretation of the micro-kinetics could be obtained.

1.4.3. Effectiveness Factor

The relative effectiveness factor was defined as the ratio of the reaction rate observed for the films compared to the reaction rate obtained for the powder. To validate the reaction system and measurement method, an analogous commercial catalyst (STREM n. 46-1903, loading Pd 5wt%) was used. The Basic Autoclave Equation (BAE) was used to identify conditions where reactions could be run without obvious gas/liquid mass transfer limitations (Figure 2). The global reaction rate was monitored as a function of catalyst mass loading at constant reaction conditions (5 barg hydrogen, 0.03 mol/l nitrobenzene in methanol, 20 °C stirrer speed 2000 rpm) to calculate the gas absorption resistance using the BAE (right plot of Figure S.2.). The gas absorption resistance was estimated to be 1.2 s/mmol (intercept of best-fit line with ordinate).
To obtain relevant data under conditions where diffusion limitations regarding gas/liquid mass transfer could be avoided, experiments were conducted using 25 mg catalyst (1.25 mg Pd), pressures below 5 bars, and nitrobenzene concentrations below 0.03 mol/l in methanol at stirring speeds of 2000 rpm. These conditions ensured operation in a regime not limited by gas/liquid mass transfer.

A comparable activation energy (with an average of 23 kJ/mol) was calculated for the 100, 250, and 400 μm fibrillated films in the range of -8°C to 20 °C. The reaction rate decreased with increasing film thickness, resulting in a decrease in the effectiveness factor for the respective films (Figure 6). For reference, the effectiveness factor of the powder measured in the kinetic regime was normalized to 100%.

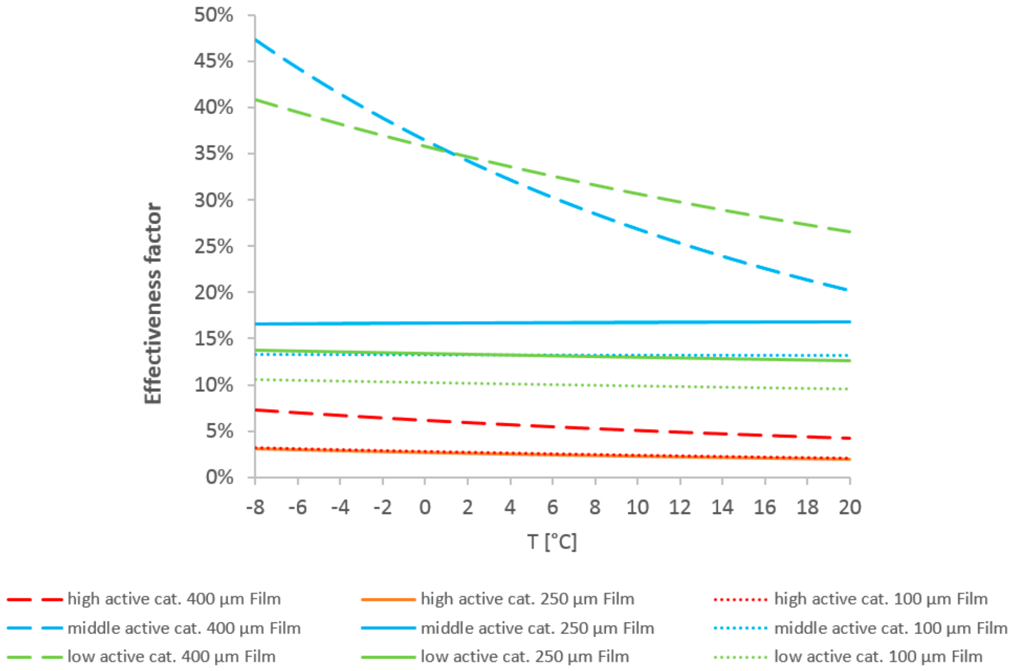

Figure S3. Effectiveness factor of the fibrillated films produced from the three Pd/C catalysts (see table 1 and 2).

In Figure S3, it can be observed that the highest active catalyst film exhibits an effectiveness factor of less than 8% at -8°C. This indicates that less than 8% of the porous body is effectively utilized, highlighting the presence of massive mass transfer limitations. For the catalyst films of middle and low activity, the effectiveness factor is higher (ranging from 40% to 47% for the 100 µm film, as shown in Figure 6), but still far from 100% assigned to the free-flowing powder. Different strategies to optimize the limitations of the catalyst films will be described in the following paragraph.

1.4.4 Flow reactor experiments

For the flow reactor tests, the fibrillated films were immobilized on the micro-channels of a 100 µl microreactor and tested in continuous mode. The film was gently pressed into the microstructures, forming one of the walls of the channel. The catalyst performance over time was monitored using the hydrogenation of nitrobenzene as a test reaction.

A nitrobenzene solution (0.03 mol/l in methanol, liquid flow rate of 2 ml/min) was supplied by a syringe pump. This solution was mixed with hydrogen (approximately in a volume ratio of 1:5) using a T junction and then inserted into the microreactor. The reaction was carried out at

20 °C and atmospheric pressure, with an approximate residence time of about 5 seconds. The evaluation of the product was determined via UV-Vis and GC-MS analysis.
